# The Role of Androgen Receptor and Antiandrogen Therapy in Breast Cancer: A Scoping Review

**DOI:** 10.3390/curroncol33010041

**Published:** 2026-01-12

**Authors:** Antonio Ghidini, Roberta Bukovec, Luisa Roncari, Isabella Garassino, Fulvia Milena Cribiù, Fausto Petrelli

**Affiliations:** 1 Oncology Unit, Casa di Cura Igea, 20129 Milano, MI, Italy; a.ghidini@casadicuraigea.it (A.G.); r.bukovec@casadicuraigea.it (R.B.); luisa.roncari@alice.it (L.R.); isabella.garassino@virgilio.it (I.G.); 2 Patology Unit, ASST Bergamo Ovest, 24047 Treviglio, BG, Italy; fulvia_cribiu@asst-bgovest.it; 3 Oncology Unit, ASST Bergamo Ovest, 24047 Treviglio, BG, Italy

**Keywords:** androgen receptor, triple-negative breast cancer, luminal androgen receptor, antiandrogen therapy, enzalutamide, bicalutamide, hormone receptors, precision oncology

## Abstract

Breast cancer is a complex and highly heterogeneous disease, and its management is increasingly moving towards the principles of precision medicine. In this context, the androgen receptor (AR) has emerged as a promising therapeutic target, particularly within the challenging subgroup of triple-negative breast cancers (TNBCs) that express it. This scoping review provides a comprehensive and detailed analysis of the multifaceted role of AR in breast cancer. We delve into its intricate molecular structure, its differential function in ER-positive vs. TNBC subtypes, and the detailed molecular mechanisms that govern its activity. We provide a thorough examination of the landmark clinical trials with antiandrogen agents, including not only enzalutamide but also other first- and second-generation compounds, and discuss the emerging data on their efficacy. Furthermore, we will explore the critical challenges that hinder their widespread clinical adoption, such as primary and acquired resistance mechanisms, the need for robust predictive biomarkers, and the heterogeneity of AR expression. Finally, we outline future research directions, focusing on novel combination therapies and the development of next-generation agents and predictive tools to optimize patient selection and improve clinical outcomes.

## Simple Summary

Breast cancer is not one disease but many different types that behave differently and need different treatments. Most therapies target the female hormones, estrogen and progesterone, but another hormone receptor, called the androgen receptor, is also found in many breast cancers, including some aggressive tumors that lack the usual treatment targets. This review summarizes what is currently known about how the androgen receptor affects breast cancer development and how medicines that block it may help certain patients. We describe how the androgen receptor works inside cells, why it may slow tumor growth in some breast cancers but support growth in others, and what clinical studies have reported so far for antiandrogen drugs. Overall, benefits have generally been modest, and responses appear concentrated in carefully selected patients, meaning these treatments are not yet routine care. We also explain why resistance can occur and why better laboratory tests are needed to identify who is most likely to benefit. Finally, we outline future directions, including combining androgen receptor-blocking drugs with other treatments and using modern blood- and tissue-based testing to personalize therapy over time. This work is valuable because it may guide the development of new options for patients with limited choices while helping avoid ineffective treatment.

## 1. Introduction

The molecular classification of breast cancer, pioneered by landmark gene expression profiling studies, has fundamentally reshaped our understanding of the disease, moving beyond simple histopathological classifications. The current molecular classification, based on the expression of the estrogen receptor (ER), the progesterone receptor (PR), and the human epidermal growth factor receptor 2 (HER2), has allowed for effective patient stratification into clinically distinct subtypes: luminal A, luminal B, HER2-enriched, and basal-like [1]. However, the triple-negative breast cancer (TNBC) subtype, defined by the absence of all three markers, remains one of the most formidable therapeutic challenges, accounting for approximately 15–20% of all breast cancer cases [2].

TNBC is not a single entity; rather, it is a highly heterogeneous group of tumors with diverse molecular and clinical characteristics. Gene expression profiling has further subdivided TNBC into several distinct subtypes, including basal-like 1 (BL1), basal-like 2 (BL2), immunomodulatory (IM), mesenchymal (M), mesenchymal stem-like (MSL), and luminal androgen receptor (LAR) [3]. This intrinsic heterogeneity explains why a single therapeutic approach, such as conventional chemotherapy, often yields varied and unpredictable responses. Due to its high aggressiveness, a tendency for early recurrence, and a lack of established targeted therapies, TNBC has a significantly more unfavorable prognosis compared to other subtypes. In this challenging landscape, the identification of novel, actionable therapeutic targets is essential to improving patient outcomes.

The AR, a nuclear hormone receptor, has emerged as one such target. While its role in prostate cancer is well-established as a primary driver, its function in breast cancer is complex and context-dependent. AR is expressed in a large majority of ER-positive tumors (70–90%) and, more importantly, in a significant fraction (12–55%) of TNBCs [4]. Its expression in this historically “untargetable” subtype has positioned AR at the center of intense research, offering a new potential avenue for personalized therapy.

The objective was to map the extent, range, and nature of evidence regarding the role of the AR and antiandrogen therapy in breast cancer, without restricting inclusion to specific study designs or outcome measures.

## 2. Methods

### 2.1. Protocol and Framework

This scoping review was conducted according to the methodological framework proposed by Arksey and O’Malley and subsequent refinements, and was informed by guidance from the Joanna Briggs Institute (JBI). Reporting was guided by the PRISMA-ScR checklist to enhance transparency and reproducibility [4,5,6,7]. The protocol for this scoping review was registered with Figshare and is available at https://figshare.com/articles/journal_contribution/_p_dir_ltr_b_The_Role_of_Androgen_Receptor_and_Antiandrogen_Therapy_in_Breast_Cancer_A_Scoping_Review_b_p_/31032145?file=60885565 (accessed on 4 January 2026).

### 2.2. Objectives

The primary objective of this scoping review was to map the available evidence on the biological role of the androgen receptor and the therapeutic relevance of antiandrogen agents in breast cancer, with a particular focus on triple-negative and LAR subtypes. Secondary aims were to identify existing knowledge gaps, ongoing clinical trials, and emerging therapeutic strategies involving AR modulation.

### 2.3. Eligibility Criteria

Studies were eligible if they met at least one of the following criteria:

- Investigated the expression, biological function, or prognostic significance of AR in breast cancer;
- Evaluated clinical outcomes of antiandrogen therapy (e.g., enzalutamide, bicalutamide, apalutamide, darolutamide);
- Provided mechanistic or translational insights into AR signaling in breast tumor biology.

All study designs (preclinical, translational, and clinical) were considered. Only articles published in English were included. Editorials, single case reports, and non–peer-reviewed materials were excluded.

### 2.4. Information Sources and Search Strategy

**Figure 1:**
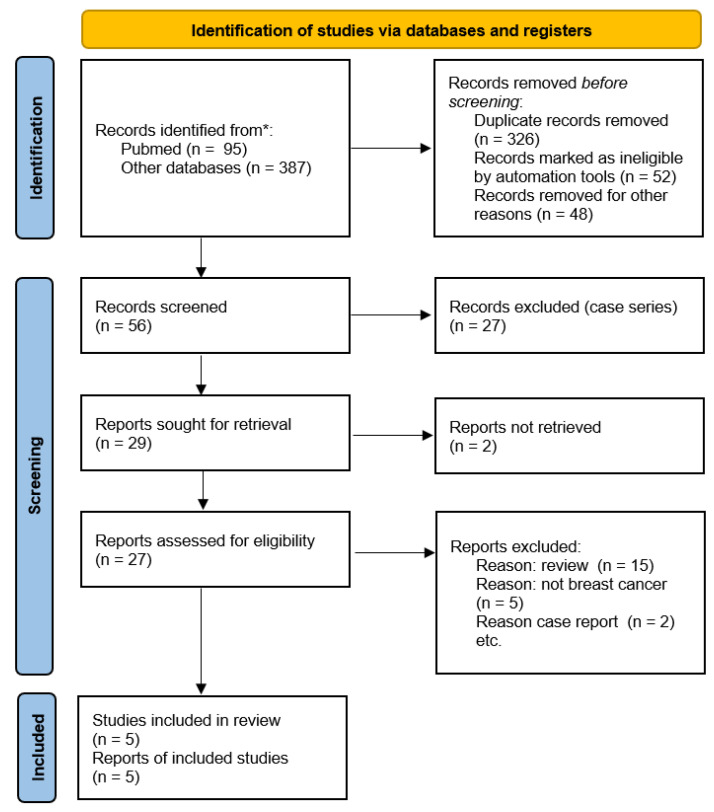
Flow diagram of included studies.

A comprehensive literature search was performed in PubMed/MEDLINE, Scopus, and ClinicalTrials.gov up to 23 December 2025. Search strategies combined Medical Subject Headings (MeSH) and free-text terms related to AR, breast cancer, and antiandrogen therapy (e.g., “androgen receptor”, “breast neoplasms”, “enzalutamide”, “bicalutamide”, “apalutamide”, “luminal AR”, “triple-negative breast cancer”). Reference lists of relevant reviews and primary studies were manually screened to identify additional eligible records. ClinicalTrials.gov was also queried on 23 December 2025 to identify registered interventional studies of AR-targeted therapy in breast cancer, including ongoing or recently completed trials without mature peer-reviewed efficacy results (Figure 1).

### 2.5. Selection Process

Two reviewers independently screened titles and abstracts for relevance. Full texts of potentially eligible studies were then assessed against the inclusion criteria. Any disagreements were resolved through discussion and consensus. The study selection process was documented using a PRISMA-ScR flow diagram (Figure 1).

### 2.6. Data Charting and Synthesis

Data were extracted using a standardized charting form that captured study characteristics (year of publication, study design, population, interventions, and key outcomes) and thematic focus (molecular mechanisms, prognostic implications, therapeutic trials, or resistance mechanisms). Extracted data were synthesized descriptively and thematically to highlight patterns and trends across preclinical and clinical evidence. No formal risk-of-bias assessment or quantitative meta-analysis was undertaken, in line with the exploratory purpose of a scoping review.

### 2.7. Ethical Considerations

As this review used only previously published data, ethical approval and informed consent were not required.

## 3. The AR: Structure, Function, and Pleiotropic Role in Breast Cancer (Figure 2a,b)

### 3.1. The Molecular Architecture and Mechanism of Action of the AR

The AR is a member of the steroid nuclear receptor superfamily, a class of ligand-regulated transcription factors. The AR protein comprises three major functional domains:

- The N-terminal domain (NTD): this is the largest and most variable domain. It contains the activation function 1 (AF1) region, which is critical for transcriptional activation and interacts with various co-activators.
- The DNA-binding domain (DBD): this highly conserved domain is responsible for recognizing and binding to specific DNA sequences known as Androgen Response Elements (AREs) in the promoter regions of target genes.
- The ligand-binding domain (LBD): located at the C-terminus, this domain binds to endogenous ligands, primarily testosterone and its more potent metabolite, dihydrotestosterone (DHT).

**Figure 2:**
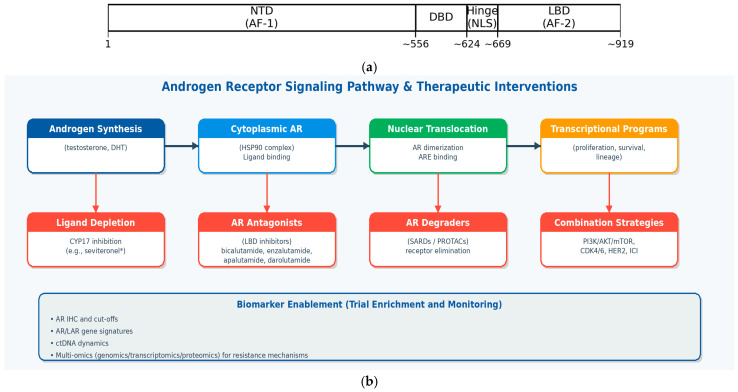
(**a**). Schematic representation of the androgen receptor protein domains (approximate), including the N-terminal domain (NTD; AF-1), DNA-binding domain (DBD), hinge region (containing nuclear localization signals, NLS), and ligand-binding domain (LBD; AF-2). (**b**). Therapeutic targeting of the androgen receptor axis in breast cancer. Multiple intervention points are clinically or preclinically tractable, including ligand depletion, AR antagonism at the ligand-binding domain, and direct receptor degradation; biomarker strategies (including AR IHC, gene signatures, ctDNA, and multi-omics) may enable patient enrichment and real-time monitoring of resistance.

In its inactive state, the AR resides in the cytoplasm, complexed with chaperone proteins like heat shock protein 90 (Hsp90). The binding of an androgen ligand to the LBD induces a conformational change, leading to the dissociation of Hsp90. This activation step exposes a nuclear localization signal, causing the activated receptor to undergo dimerization and subsequently translocate into the cell nucleus. Once inside the nucleus, the AR dimer binds to AREs on the DNA and recruits a large complex of co-activators (e.g., p300/CBP, NCOA1) or co-repressors that collectively regulate the transcription of target genes [8].

Canonical (genomic) AR signaling is initiated by ligand binding (testosterone or dihydrotestosterone), followed by receptor dimerization and nuclear translocation. In the nucleus, AR binds androgen response elements (AREs) and recruits co-regulators to modulate transcriptional programs that can influence proliferation, survival, differentiation, and metabolic reprogramming in a context-dependent manner [9,10,11].

In addition, AR can engage non-genomic signaling through cytoplasmic interactions with kinases and adaptor proteins, including crosstalk with Phosphoinositide 3-kinase/Protein kinase B (PKB)/Mammalian target of rapamycin and Mitogen-activated protein kinase (PI3K/AKT/mTOR and MAPK) pathways. Such pathway convergence is particularly relevant in TNBC, where co-dependencies (e.g., frequent PI3K pathway alterations in the LAR subtype) provide a biological rationale for combination strategies [9,11,12,13].

Therapeutic inhibition of the AR axis can be achieved at multiple nodes, including ligand depletion, competitive antagonism at the ligand-binding domain (LBD), inhibition of AR nuclear translocation and DNA binding, and direct receptor degradation (e.g., selective AR degraders and PROTAC-based approaches). Clinically, the most mature strategy in breast cancer has been LBD antagonism with first- and second-generation antiandrogens (including bicalutamide, enzalutamide, apalutamide, and darolutamide), while AR degraders represent a rapidly evolving class with the potential to address some resistance mechanisms [9,11,14].

### 3.2. The Context-Dependent Role of AR in Breast Cancer Subtypes

The function of the AR in breast cancer is not uniform; it is a pleiotropic actor whose role is dictated by the specific molecular context of the tumor.

A. Biological Function in ER-positive Luminal Cancer: in the majority of luminal tumors, AR appears to have a protective or tumor-suppressive role [7]. Numerous preclinical and clinical studies have shown that AR expression is correlated with a better prognosis, lower tumor grade, and improved response to conventional endocrine therapies [9]. This apparently paradoxical function results from a complex and well-documented crosstalk between the AR and ER signaling pathways. Several mechanisms have been proposed to explain this interaction:

- Transcriptional Interference: AR can physically interact with ER, leading to a direct inhibition of ER-mediated transcriptional activity.
- Competition for Co-regulators: AR and ER compete for shared co-activators, such as AIB1 (NCOA3), leading to a reduction in the transcriptional output of the more proliferative ER pathway.
- Substrate Level Competition: Both AR and ER can respond to certain steroid precursors, leading to competition for the available ligands within the tumor microenvironment.
- Regulation of Aromatase: The AR pathway may also influence the expression or activity of aromatase, the key enzyme in estrogen synthesis, thereby indirectly modulating the ER signal. This complex interplay partly explains why some ER+/AR+ tumors respond better to conventional endocrine therapies like tamoxifen or aromatase inhibitors [8].

Collectively, these models support a largely context-dependent antagonism between AR and ER signaling in many luminal tumors, but highlight that the direction and magnitude of AR/ER crosstalk can vary with receptor stoichiometry, ligand milieu, and co-regulator availability. This biological plasticity likely contributes to the heterogeneous prognostic associations reported for AR in ER-positive disease and provides a strong rationale for biomarker-driven trial designs rather than unselected clinical testing [9,11,15,16,17].

B. Biological Function in TNBC: AR expression is enriched in the luminal AR (LAR) or ‘molecular apocrine’ subtype, which is characterized by a luminal-like transcriptional program, apocrine differentiation, and frequent alterations in the PI3K pathway. In this context, AR can function as a lineage-associated driver, and pharmacologic AR inhibition has demonstrated anti-tumor activity in preclinical models, providing a clear therapeutic rationale for biomarker-selected clinical trials in AR-positive TNBC [7,9,10,11,12].

## 4. In-Depth Analysis of Clinical Trials with Antiandrogens: A Critical Examination

The identification of AR as a druggable target has led to a growing portfolio of phase I–II clinical studies testing antiandrogens across distinct breast cancer phenotypes. Overall, monotherapy activity has been modest and appears concentrated in biomarker-selected subsets (particularly AR-positive TNBC/LAR and selected HER2-positive disease), supporting the need for refined biomarker strategies and rational combinations. A summary of key completed/published trials and selected registered studies without mature peer-reviewed efficacy results is provided in Table 1 and Table 2.

### 4.1. The Enzalutamide Trials: A Turning Point

A. MDV3100-11 (NCT01889238) [18]: This open-label, Simon two-stage phase II study evaluated enzalutamide monotherapy in patients with locally advanced or metastatic AR-positive TNBC (AR positivity defined by nuclear AR staining by IHC). Enzalutamide demonstrated modest but clinically meaningful activity in a biomarker-selected subset with a manageable safety profile. Exploratory analyses suggested that an AR-related transcriptional signature may further enrich for clinical benefit, underscoring the importance of molecular selection beyond IHC alone.

B. Fulvestrant plus enzalutamide (NCT02953860) [19]: This phase II study evaluated combined ER and AR blockade in metastatic ER-positive/HER2-negative breast cancer. Dual pathway inhibition provided clinical benefit in a subset of patients, including in endocrine-resistant settings, and supports the mechanistic premise that AR/ER crosstalk can be therapeutically leveraged. Future work should clarify optimal patient selection (e.g., AR thresholds, AR-driven transcriptional programs, and co-alterations such as PI3K pathway changes).

C. Exemestane with or without enzalutamide (NCT02007512) [20]: This randomized, placebo-controlled phase II trial tested whether adding enzalutamide to exemestane improves outcomes in HR-positive/HER2-normal advanced breast cancer. While the overall benefit appeared limited in unselected populations, the study provides a clinically informative benchmark for dual ER/AR pathway inhibition and highlights the need for predictive biomarkers and combination strategies.

D. Enzalutamide plus trastuzumab (NCT02091960) [21]: This phase II trial evaluated enzalutamide combined with trastuzumab in patients with HER2-positive, AR-positive locally advanced or metastatic breast cancer. The regimen was generally well tolerated and showed clinical activity in selected patients, supporting ongoing efforts to integrate AR targeting into HER2-directed strategies with biomarker optimization.

### 4.2. Studies with Bicalutamide and Other Antiandrogens

A. Bicalutamide (TBCRC 011; NCT00468715) [22]: This pivotal single-arm phase II study evaluated the first-generation antiandrogen bicalutamide in AR-positive, ER/PR-negative metastatic breast cancer. While objective responses were uncommon, a subset of patients achieved durable disease stabilization, providing early proof-of-principle that AR can represent a therapeutically actionable dependency in selected TNBC/molecular apocrine tumors.

B. Apalutamide and darolutamide: newer, potent second-generation AR antagonists with improved pharmacologic profiles have generated interest for breast cancer translation. Multiple biomarker-selected studies are registered in AR-positive TNBC (e.g., darolutamide studies such as NCT03383679), and additional trials are exploring AR inhibition in combination with pathway inhibitors or chemotherapy. Although peer-reviewed efficacy data remain limited, these studies will help define whether next-generation AR antagonists offer incremental benefit over earlier agents and which molecular contexts are most sensitive [9,11].

**Table 1:** Completed and published clinical studies of androgen receptor (AR) targeting in breast cancer (antiandrogen monotherapy or combination therapy). Abbreviations: AR, androgen receptor; IHC, immunohistochemistry; TNBC, triple-negative breast cancer; HR, hormone receptor; CBR, clinical benefit rate.

Trial (Acronym; Registry ID)	Population/Setting	AR Definition	Intervention	Phase/Design	Key Outcomes (As Reported)
TBCRC 011; NCT00468715	Metastatic ER/PR-negative breast cancer (molecular apocrine phenotype)	AR+ by IHC (commonly ≥10% nuclear staining)	Bicalutamide 150 mg daily	Phase II, single-arm	CBR at 6 months: 19% (single-arm phase II); objective responses uncommon; tolerability acceptable [22].
MDV3100-11; NCT01889238	Locally advanced/metastatic AR-positive TNBC	Nuclear AR staining by IHC (threshold per assay/definition in trial)	Enzalutamide 160 mg daily	Phase II, Simon two-stage	CBR at 16 weeks: 25% (all enrolled) and 33% (evaluable); median OS reported ~12.7 months (all) and 17.6 months (evaluable) [18].
NCT02953860	Metastatic ER-positive/HER2-negative breast cancer (endocrine-resistant enriched)	Protocol-defined (AR assessed and biopsies included)	Fulvestrant + enzalutamide	Phase II	Clinical benefit observed in a subset; paired biopsies support biomarker discovery and pathway interrogation [19].
NCT02007512	Advanced HR-positive/HER2-normal breast cancer (postmenopausal cohorts)	Exploratory AR assessment (subgroup analyses reported)	Exemestane + enzalutamide vs. exemestane + placebo	Randomized, placebo-controlled phase II	Overall benefit limited in unselected populations; highlights need for predictive biomarkers and rational combinations [20]
NCT02091960	Locally advanced/metastatic HER2-positive, AR-positive breast cancer	AR+ by IHC (per trial assay)	Trastuzumab + enzalutamide	Phase II, single-arm	Clinical activity in selected patients; generally well tolerated; biomarker refinement needed [21]

**Table 2:** Selected registered clinical trials of AR-targeted therapy in breast cancer without mature peer-reviewed efficacy results at the time of writing (registry-derived; trial status and details should be verified on the registry prior to use). Abbreviations: CDK4/6, cyclin-dependent kinase 4/6.

Registry ID	Subtype/Setting (Selection)	Intervention	Phase/Design	Notes (Biomarkers/Rationale)
NCT03383679	AR-positive TNBC	Darolutamide (monotherapy)	Phase II	Second-generation AR antagonist; contributes evidence on activity/tolerability in AR-positive TNBC.
NCT02689427	Stage I–III AR-positive TNBC (neoadjuvant)	Enzalutamide + paclitaxel (pre-surgery)	Phase IIB	Explores chemo-sensitization and early pharmacodynamic endpoints; opportunity for tissue-based biomarker analysis.
NCT02457910	Advanced breast cancer with AR pathway dependence (protocol-defined)	Taselisib + enzalutamide	Phase I/II	Targets PI3K and AR co-dependency observed in LAR biology; tests pathway-combination hypothesis.
NCT03207529	Advanced breast cancer (protocol-defined; PI3K pathway focus)	Alpelisib + enzalutamide	Phase I/II	PI3K-alpha inhibition combined with AR blockade; aligns with frequent PI3K alterations in LAR tumors.
NCT04142060	HR-positive/HER2-negative breast cancer (PAM50 HER2-enriched subset)	Enzalutamide (window/biomarker-driven)	Phase II	Evaluates AR pathway modulation in transcriptionally HER2-enriched tumors; biomarker enrichment strategy.
NCT02910050	Metastatic ER-positive/AR-positive breast cancer	Aromatase inhibitor-based endocrine therapy + bicalutamide	Phase II	Tests whether AR blockade augments endocrine therapy; addresses AR/ER crosstalk in luminal disease.
NCT02749903	Advanced AR-positive breast cancer (protocol-defined)	Enzalutamide-based therapy	Phase II	Additional registry-identified enzalutamide study; population/selection depends on protocol specifics.
NCT06099769	Breast cancer with AR pathway dependence (protocol-defined)	Enzalutamide alone vs. enzalutamide + mifepristone	Phase II	Evaluates whether additional hormonal modulation improves efficacy; integrates endocrine pathway crosstalk.
NCT06365788	Locally advanced unresectable or metastatic AR-positive TNBC	Bicalutamide + abemaciclib	Phase I/II	Combination of AR blockade with CDK4/6 inhibition; tests cell-cycle synergy in AR-positive disease.

## 5. Understanding Resistance: Primary and Acquired Mechanisms

Despite the clinical benefit observed in a subset of patients, many tumors are either intrinsically resistant to antiandrogen therapy or develop resistance over time. Understanding these mechanisms is crucial for developing new therapeutic strategies.

### 5.1. Primary (Intrinsic) Resistance

AR-independent Proliferation: many TNBC subtypes, particularly non-LAR variants, do not depend on the AR pathway for their growth. In these tumors, AR may be expressed, but it is not a “driver” gene. The tumor’s proliferation is sustained by other oncogenic pathways (e.g., epidermal growth factor receptor (EGFR), PI3K/AKT/mTOR, RAS/MAPK), rendering antiandrogens ineffective. This underscores the urgent need for robust predictive biomarkers that can distinguish LAR from non-LAR subtypes [9,10,11,23].

Low AR Expression: tumors with very low AR expression levels (e.g., <10% on IHC) are unlikely to be responsive to antiandrogen therapy, as the signal is not sufficiently strong to sustain tumor growth [18,22].

Aberrant AR Signaling: some tumors may have AR signaling but a different downstream effector profile, or they may be regulated by different co-activators, making them less sensitive to standard AR inhibitors [9,11,23,24].

### 5.2. Acquired (Secondary) Resistance

AR Splice Variants: the most well-studied mechanism of acquired resistance is the development of AR splice variants, such as AR-V7. These truncated forms of the AR protein lack the LBD but retain the NTD and DBD. Consequently, they are constitutively active and can drive gene expression without the need for an androgen ligand, making them completely resistant to antiandrogen drugs that target the LBD (like enzalutamide and apalutamide) [23,24,25].

AR Ligand-Binding Domain Mutations: mutations in the LBD can alter the receptor’s affinity for its ligand or for the antiandrogen, leading to drug resistance. Some mutations can even turn antiandrogens into agonists, paradoxically activating the AR pathway [26].

Activation of Compensatory Pathways: tumors can evade antiandrogen therapy by activating alternative signaling pathways that compensate for AR inhibition. The PI3K/AKT/mTOR pathway is a prime example. This pathway is frequently activated in AR-positive breast cancers and can be a major driver of resistance, providing a strong rationale for combination therapies [12]. Other pathways, such as MAPK, can also be activated.

Tumor Microenvironment (TME) Changes: the TME plays a significant role in therapeutic response. Changes in the composition of the TME, including the recruitment of immune cells or fibroblasts, can create a pro-proliferative and anti-apoptotic environment that bypasses the need for AR signaling [27,28].

## 6. Therapeutic Synergies and Future Perspectives

Given the suboptimal efficacy of antiandrogens as monotherapy, the future of this approach lies in combination strategies designed to overcome resistance and enhance efficacy.

### 6.1. Combination with Signaling Pathway Inhibitors

AR + PI3K/AKT/mTOR Pathway Inhibitors: this is one of the most promising combinations. Preclinical data show that co-inhibition of AR and the PI3K/AKT/mTOR pathway leads to a synergistic effect on tumor growth inhibition and cell death. Clinical trials evaluating combinations like enzalutamide with mTOR inhibitors (e.g., everolimus) are underway, aiming to simultaneously block multiple growth signals and prevent the activation of bypass pathways. AR + CDK4/6 Inhibitors: cyclin-dependent kinases 4 and 6 (CDK4/6) are key regulators of the cell cycle, and their inhibitors (e.g., palbociclib, ribociclib) have revolutionized the treatment of ER-positive breast cancer. Given the AR-driven proliferation in LAR tumors, a combination with a CDK4/6 inhibitor is a logical strategy to block cell cycle progression and is currently being investigated in clinical settings [13,29,30,31,32].

### 6.2. Combination with Chemotherapy

Preclinical evidence suggests that AR signaling can mediate resistance to certain chemotherapeutic agents, particularly taxanes. By blocking AR, antiandrogens may re-sensitize tumor cells to standard chemotherapy, making the combination synergistic. Such combinations could benefit patients with advanced, chemotherapy-resistant disease.

### 6.3. Combination with Immunotherapy

The AR axis has recently been implicated in modulating the tumor microenvironment and the immune response. AR signaling can influence the expression of immune checkpoint ligands like PD-L1 and the infiltration of immune cells into the tumor. Blocking AR could therefore enhance the response to immune checkpoint inhibitors (ICIs) such as anti-PD-1 or anti-PD-L1 antibodies [27]. This represents an especially active and promising area of translational research, as it could open up new treatment possibilities for TNBC, a tumor type that is already a candidate for immunotherapy.

### 6.4. Novel Agents and Approaches

The development of new therapeutic agents is also a key part of the future landscape. AR degraders, or PROTACs, are a new class of molecules designed to not only inhibit but also actively degrade the AR protein. These agents hold the potential to overcome resistance mechanisms like AR splice variants by completely removing the target protein from the cell [14].

## 7. Towards a New Era of Precision Therapy: The Critical Need for Biomarkers

A key obstacle to the broader adoption of antiandrogen therapy to the widespread adoption of antiandrogen therapy in breast cancer is the lack of a reliable, universally accepted predictive biomarker.

### 7.1. The Limitations of Immunohistochemistry (IHC)

While IHC for AR expression is the most common method for patient selection in clinical trials, it has several limitations. The cut-off value for what constitutes “AR-positive” (e.g., ≥1% vs. ≥10%) is not standardized, leading to inconsistencies. Furthermore, IHC measures only the presence of the protein, not its functional activity or its role as a driver of the tumor’s growth. This explains why many AR-positive tumors on IHC do not respond to antiandrogen therapy.

### 7.2. The Promise of Gene Expression Signatures

The AR-gene signature (AR-GS), first used in the Gucalp trial, represents a significant step forward. By measuring the expression of a panel of genes that are regulated by a functionally active AR, this signature can better identify tumors that are truly dependent on the androgen pathway. Gene expression signatures, such as the luminal-AR subtype within the TNBC classification, hold greater promise than simple protein expression for predicting therapeutic response.

### 7.3. The Future: Multi-Omics and Liquid Biopsies

Future biomarker development will likely integrate multi-omics data (genomics, transcriptomics, epigenomics, and proteomics) to define AR dependency states, identify co-alterations that shape pathway crosstalk (e.g., PI3K pathway changes), and characterize resistance biology. In parallel, liquid biopsy technologies—particularly circulating tumor DNA (ctDNA)—provide a minimally invasive means for longitudinal monitoring. ctDNA kinetics can offer early indications of response, while emergent genomic alterations may be detectable before radiographic progression, enabling adaptive trial designs and more timely treatment switching. A contemporary approach will likely combine tissue-based multi-omics with serial ctDNA profiling to refine patient enrichment, track clonal evolution, and prioritize rational combinations for AR-targeted therapy [33,34,35,36,37,38,39,40,41,42].

## 8. Conclusions

The journey of antiandrogen therapy in breast cancer represents a compelling case study in the evolution of precision medicine. From a simple observation of AR expression, we have moved to a sophisticated understanding of its complex, context-dependent role in various tumor subtypes. Although early trials with enzalutamide monotherapy demonstrated only modest activity, they provided compelling proof that the AR is a valid and druggable therapeutic target, particularly in a subset of TNBCs. The challenges posed by resistance and disease heterogeneity underscore the need for a more nuanced, biology-driven approach. The future of this field lies not in a one-size-fits-all solution but in a multi-pronged strategy that includes:

- The development of reliable predictive biomarkers that can precisely identify the patients who will benefit most.
- The optimization of combination therapies that target multiple, synergistic pathways to overcome resistance.
- The exploration of next-generation agents that can overcome existing resistance mechanisms, such as AR splice variants.

Through integration of these strategies, we can move closer to the goal of offering a truly personalized and effective therapy for patients with AR-positive TNBC, a tumor subtype that has long been without targeted options. This shift is a powerful testament to how a deeper understanding of molecular biology can transform the landscape of cancer treatment and offer new hope to patients.

## Data Availability

No new data were created or analyzed in this study.

## References

[1] Perou C.M., Sørlie T., Eisen M.B., van de Rijn M., Jeffrey S.S., Rees C.A., Pollack J.R., Ross D.T., Johnsen H., Akslen L.A. (2000). Molecular portraits of human breast tumours. Nature.

[2] Dent R., Trudeau M., Pritchard K.I., Hanna W.M., Kahn H.K., Sawka C.A., Lickley L.A., Rawlinson E., Sun P., Narod S.A. (2007). Triple-negative breast cancer: Clinical features and patterns of recurrence. Clin. Cancer Res..

[3] Lehmann B.D., Bauer J.A., Chen X., Sanders M.E., Chakravarthy A.B., Shyr Y., Pietenpol J.A. (2011). Identification of human triple-negative breast cancer subtypes and preclinical models for selection of targeted therapies. J. Clin. Investig..

[4] Arksey H., O’Malley L. (2005). Scoping studies: Towards a methodological framework. Int. J. Soc. Res. Methodol..

[5] Levac D., Colquhoun H., O’Brien K.K. (2010). Scoping studies: Advancing the methodology. Implement. Sci..

[6] Peters M.D.J., Godfrey C., McInerney P., Munn Z., Tricco A.C., Khalil H. (2020). Chapter 11: Scoping Reviews. JBI Manual for Evidence Synthesis.

[7] Tricco A.C., Lillie E., Zarin W., O’Brien K.K., Colquhoun H., Levac D., Moher D., Peters M.D.J., Horsley T., Weeks L. (2018). PRISMA extension for scoping reviews (PRISMA-ScR): Checklist and explanation. Ann. Intern. Med..

[8] Pratt W.B., Toft D.O. (1997). Steroid receptor interactions with heat shock protein and immunophilin chaperones. Endocr. Rev..

[9] Dai C., Ellisen L.W. (2023). Revisiting Androgen Receptor Signaling in Breast Cancer. Oncologist.

[10] McNamara K.M., Yoda T., Takagi K., Miki Y., Suzuki T., Sasano H. (2013). Androgen receptor in triple negative breast cancer. J. Steroid Biochem. Mol. Biol..

[11] Zhao J., Huang L., Zhang X., Li Y., Zhu L., Liu Y., Zhao S., Zhao Y. (2020). Androgen Receptor in Breast Cancer: From Bench to Bedside. Front. Endocrinol..

[12] Pungsrinont T., Kallenbach J., Baniahmad A. (2021). Role of PI3K-AKT-mTOR Pathway as a Pro-Survival Signaling and Resistance-Mediating Mechanism to Therapy of Prostate Cancer. Int. J. Mol. Sci..

[13] Gordon M.A., D’Amato N.C., Gu H., Babbs B., Wulfkuhle J., Petricoin E.F., Gallagher I., Dong T., Torkko K., Liu B. (2017). Synergy Between Androgen Receptor Antagonism and Inhibition of mTOR and HER2 in Breast Cancer. Mol. Cancer Ther..

[14] Zhao L., Han X., Lu J., McEachern D., Rozovskaia T., Rychahou P., Yu S., Wang S. (2020). A highly potent PROTAC androgen receptor (AR) degrader ARD-61 effectively inhibits AR-positive breast cancer cell growth in vitro and tumor growth in vivo. Neoplasia.

[15] Hickey T.E., Selth L.A., Chia K.M., Laven-Law G., Milioli H.H., Roden D., Jindal S., Hui M., Finlay-Schultz J., Ebrahimie E. (2021). The androgen receptor is a tumor suppressor in estrogen receptor-positive breast cancer. Nat. Med..

[16] Zhong W., Yi J., Wu H., Cai Y., Huang Y., Guan X. (2022). Androgen receptor expression and its prognostic value in T1N0 luminal/HER2- breast cancer. Future Oncol..

[17] Khan A.F., Karami S., Peidl A.S., Zubair T., Merenbakh-Lamin K., Boldt H.B., Ben-Baruch N., Wolf I., Korach J., Zick A. (2023). Androgen Receptor in Hormone Receptor-Positive Breast Cancer. Int. J. Mol. Sci..

[18] Traina T.A., Miller K., Yardley D.A., Eakle J., Schwartzberg L.S., O’Shaughnessy J., Gradishar W., Schmid P., Winer E., Kelly C. (2018). Enzalutamide for the Treatment of Androgen Receptor-Expressing Triple-Negative Breast Cancer. J. Clin. Oncol..

[19] Elias A.D., Spoelstra N.S., Staley A.W., Kabos P., Sartorius C.A., Oesterreich S., Richer J.K. (2023). Phase II trial of fulvestrant plus enzalutamide in ER+/HER2− advanced breast cancer. NPJ Breast Cancer.

[20] Krop I., Abramson V., Colleoni M., Traina T., Holmes F., García-Estévez L., Hart L., Awada A., Zamagni C., Campone M. (2020). A Randomized Placebo Controlled Phase II Trial Evaluating Exemestane With or Without Enzalutamide in Patients with Hormone Receptor-Positive Breast Cancer. Clin. Cancer Res..

[21] Wardley A., Cortes J., Provencher L., Miller K., Chien A.J., Rugo H.S., Steinberg J., Sohn J., Waks A.G., O’Shaughnessy J. (2021). The efficacy and safety of enzalutamide with trastuzumab in patients with HER2+ and androgen receptor-positive metastatic or locally advanced breast cancer. Breast Cancer Res. Treat..

[22] Gucalp A., Tolaney S., Isakoff S.J., Ingle J.N., Liu M.C., Carey L.A., Blackwell K., Rugo H., Nabell L., Forero A. (2013). Phase II trial of bicalutamide in patients with androgen receptor-positive, estrogen receptor-negative metastatic Breast Cancer. Translational Breast Cancer Research Consortium (TBCRC 011). Clin. Cancer Res..

[23] Tien A.H., Sadar M.D. (2024). Treatments Targeting the Androgen Receptor and Its Splice Variants in Breast Cancer. Int. J. Mol. Sci..

[24] Bonnefoi H., Lerebours F., Pulido M., Cottu P., Sablin M.P., Frenel J.S., Dalenc F., Levy C., Mouret-Reynier M.A., Goncalves A. (2025). Darolutamide or capecitabine in triple-negative, androgen receptor-positive, advanced breast cancer (UCBG 3-06 START): A multicentre, non-comparative, randomised, phase 2 trial. Lancet Oncol..

[25] Ferguson D.C., Choi J.K., Zhu Y., Bui M.M., Coppola D., Koomen J.M., Yeh E.S. (2022). Androgen receptor splice variant-7 in breast cancer: Clinical and pathologic correlations. Mod. Pathol..

[26] Korpal M., Korn J.M., Gao X., Rakiec D.P., Ruddy D.A., Doshi S., Yuan J., Kovats S.G., Kim S., Cooke V.G. (2013). An F876L mutation in the androgen receptor mediates resistance to newer antiandrogen drugs in prostate cancer. Cancer Discov..

[27] Li P., Yuan W., Wu R., Zhu J., Jiang Y., Qin Y., Guo Y., Xu B., Zhang P. (2022). Androgens in Patients with Luminal B and HER2 Breast Cancer Might Be a Biomarker Promoting Anti-PD-1 Efficacy. Front. Oncol..

[28] Bader D.A., Chakraborty B., McDonnell D.P., Hirschey M.D. (2025). Targeting androgen receptor signaling to enhance cancer immunotherapy. Trends Pharmacol. Sci..

[29] Choupani E., Madjd Z., Saraygord-Afshari N., Nafissi N., Zarnani A.H., Roudkenar M.H., Shidfar A., Ghods R. (2022). Combination of androgen receptor inhibitor enzalutamide with the CDK4/6 inhibitor ribociclib in triple negative breast cancer cells. PLoS ONE.

[30] Lim B., Seth S., Yam C., Huo L., Fujii T., Lee J., Bassett R., Nasser S., Ravenberg L., White J. (2024). Phase 2 study of neoadjuvant enzalutamide and paclitaxel for luminal androgen receptor-enriched TNBC: Trial results and insights into “ARness”. Cell Rep. Med..

[31] Elias A.D., Staley A.W., Fornier M., Vidal G.A., Alami V., Sams S., Spoelstra N.S., Goodspeed A., Kabos P., Diamond J.R. (2024). Clinical and immune responses to neoadjuvant fulvestrant with or without enzalutamide in ER+/Her2− breast cancer. NPJ Breast Cancer.

[32] Wang Y., Liu X., Zhang H., Chen L., Zhao M., Li Y., Zhang Y., Wang J., Liu Z., Chen X. (2024). Novel degradable approach for different targets to treat breast cancer. Eur. J. Med. Chem..

[33] Cancer Genome Atlas Network (2012). Comprehensive molecular portraits of human breast tumours. Nature.

[34] Curtis C., Shah S.P., Chin S.F., Turashvili G., Rueda O.M., Dunning M.J., Speed D., Lynch A.G., Samarajiwa S., Yuan Y. (2012). The genomic and transcriptomic architecture of 2000 breast tumours reveals novel subgroups. Nature.

[35] Dawson S.J., Tsui D.W.Y., Murtaza M., Biggs H., Rueda O.M., Chin S.F., Dunning M.J., Gale D., Forshew T., Mahler-Araujo B. (2013). Analysis of circulating tumor DNA to monitor metastatic breast cancer. N. Engl. J. Med..

[36] Garcia-Murillas I., Schiavon G., Weigelt B., Ng C., Hrebien S., Cutts R.J., Cheang M., Osin P., Nerurkar A., Kozarewa I. (2015). Mutation tracking in circulating tumor DNA predicts relapse in early breast cancer. Sci. Transl. Med..

[37] Coombes R.C., Smith J., Patel A., Chen L., Gupta R., Martinez F., Ahmed S., Lee H., Johnson T., O’Connor M. (2025). Bridging the gap: ctDNA, genomics, and equity in breast cancer care. NPJ Breast Cancer.

[38] Malik S., Zaheer S. (2025). The impact of liquid biopsy in breast cancer: Redefining the landscape of non-invasive precision oncology. J. Liq. Biopsy..

[39] Magbanua M.J.M., Zhao Y., Lin Q., Roberts K.J., Singh A., Brooks D., Wang F., Ellis M.J., Crosswell A., Doyle G.V. (2025). Circulating DNA tumor fraction as a biomarker for advanced breast cancer: Analytical validity and clinical utility. Front. Oncol..

[40] Liu Y., Chen X., Zhang J., Li R., Kumar S., Gupta V., Martinez R., Zhao H., Tan W., Singh P. (2024). Artificial intelligence integrates multi-omics data for precision oncology in breast cancer. Comput. Struct. Biotechnol. J..

[41] Wang Y., Li H., Xu J., Park S., Mahmood U., Choi E., Sun T., Garcia M., Zhou L., Nguyen Q. (2025). Adaptive multi-omics integration framework for breast cancer prognosis prediction. Sci. Rep..

[42] Zhang C., Li N., Zhang P., Jiang Z., Cheng Y., Li H., Pang Z. (2024). Advancing precision and personalized breast cancer treatment through multi-omics technologies. Am. J. Cancer Res..

